# The nexus of climate and conflict in the Lake Chad Region: what we know, don’t know and need to know

**DOI:** 10.1007/s10584-025-04024-0

**Published:** 2025-10-14

**Authors:** Uche T. Okpara, Sulaiman Yunus

**Affiliations:** 1 Livelihoods and Institutions Department, Natural Resources Institute, University of Greenwich Medway Campus, Kent, ME4 4TB UK; 2 Centre for Dryland Agriculture, Bayero University, Gwarzo Road, Rimin Gata, Kano, 700281 Nigeria

**Keywords:** Climate conflict, Nexus dynamics, Anecdotal evidence, Conflict continuum, Fragility, Lake Chad region

## Abstract

Despite the substantial body of knowledge available regarding the nexus between climate and conflict, this knowledge remains scattered, fragmented and incomplete. There are various interpretations of how this nexus plays out, as well as different perspectives on the spectrum of "*climate conflict*" in fragile and conflict-affected areas. This is particularly the case in the Lake Chad region, an area that is often portrayed as a unique "testbed" for understanding climate conflict relations. This study systematically mapped and analysed published work on the nexus of climate and conflict, synthesising narratives and unpacking evidence on what we know, do not know and need to know about the nexus in the Lake Chad region. Our findings outline six key areas of knowledge that provide evidence on the nexus, including (i) how the nexus has evolved with the ongoing increase in regional climatic stress where temperatures are rising 1.5 times faster than the global average, (ii) whether studies link climatic events to different stages of the conflict cycle or the conflict continuum, and (iii) whether climatic events also introduce new forms of conflict along the conflict continuum. We note that studies failed to ask how fragility (assessed in terms of lack of state legitimacy, capacity and authority) shapes group identity/solidarity and spatiotemporal variations in climate-conflict nexus patterns and impacts. We suggest that the spectrum of what is considered "*climate conflict*" be expanded, paying particular attention to the continuum (including phases and cycles) of conflict and how different conflict types interact and reinforce one another under climate shocks. Ultimately, knowledge co-creation can help integrate fragmented evidence about the nexus, fostering a unified, coherent and verifiable body of knowledge that can support joint climate and peace initiatives and wider transformative change across the region.

**Supplementary Information:**

The online version contains supplementary material available at 10.1007/s10584-025-04024-0.

## Introduction

The scientific community has increasingly studied the nexus between climate and conflict over the past decade, indicating growing interest in the subject (Ide, 2017; Koubi, 2019; Watson *et al*., 2023). However, different conflicting perspectives exist regarding the nexus, referring to, for example, nexus dimensions, mechanisms and pathways, including diverse views on the spectrum of what ‘climate’ really means and how ‘conflict’ should be understood (Mach *et al*., 2020). Perspectives based on a combination of empirical and anecdotal evidence provide useful climate conflict lessons and suggestions for future actions (Okpara *et al*., 2018). Yet, current knowledge remains patchy, fragmented and incomplete, with some estimates and models overstating the role of climate in conflict outcomes, especially in fragile and conflict-affected areas. There have been few attempts to systematically assemble, harmonise, integrate and assess both empirical and anecdotal evidence related to the nexus. Therefore, it is crucial to prioritise the development of a well-organised, interdisciplinary knowledge base that fosters cross-sectoral learning and exchanges, while also addressing the imbalances in the production of climate-conflict knowledge.

This article is about ‘climate conflict’ evidence synthesis and analysis. The article focuses on the Lake Chad region, a typical fragile and conflict-afflicted environment that is considered by many researchers as particularly well-suited for pinning down the spectrum of what is considered a climate and conflict nexus (Vivekananda, 2018; Griffin, 2020; Jedwab et al., 2021). The Lake Chad region is threatened by high temperatures, dry air and thermal discomfort (UNEP, 2004; Okpara *et al*., 2018). Temperatures in the region are rising 1.5 times faster than the global average, spurring longer dry seasons and drying up lake ecosystems (Fisker, 2021). Temperatures are predicted to increase by 0.65 - 1.60 ^o^C and rainfall to decrease by 13–11% in the next 15 years (Climate Risk Profile: Chad, 2021). At the same time, the region is wracked by brutal conflicts (Lake Chad Basin Commission, 2018; Wakdok and Bleischwitz, 2021). Multiple conflict types occur simultaneously (Gordon, 2019), and spillover conflict dynamics have continued to reinforce conflict tipping risks, undermining regional stability (WFP, 2020; ACLED, 2022).

Previous studies (e.g., Vivekananda et al., 2019; Lamarche, 2023) have demonstrated that the relationship between climate and conflict is particularly acute in the Lake Chad region, although evidence from these studies is less harmonised and is dispersed across various platforms and institutions. Importantly, the findings are either inconclusive or the subject of much debate (Mach *et al*, 200). The scarcity of harmonised evidence raises fundamental concerns about how to assemble reliable evidence and trigger ‘quick wins’ towards cooperative adaptation and peace-centred development across the region.

This article aims to fill this gap by systematically identifying, assembling, synthesising and evaluating evidence regarding what we know, whether certain evidence is unusual, and what we do not know about climate conflict relations in the Lake Chad region. We contend that re-examining conflicting evidence on climate conflict relations in specific settings can help to reconcile current inconsistencies and discrepancies. This, in turn, can lead to a more organised, discipline-crossing and broad-based perspective and learning that can ultimately support climate resilience and peacebuilding efforts. The article identifies key areas of knowledge that provide evidence of the nexus as well as what we need to know to deepen our understanding and facilitate cross-sectoral exchanges towards unified climate and peace initiatives.

## Methodology

Following Waddington *et al*. (2012) and Okpara *et al*. (2018), we carried out a desk-based document search and literature review to unpack existing knowledge and evidence about the nexus of climate and conflict in the Lake Chad region. Desk-based document search focused on peer-reviewed and grey literature materials that contribute to and provide different forms of evidence on the nexus, covering journal articles, reviews, reports, technical papers, thesis, opinion pieces, newspaper articles and social media articles. These sources enabled us to assemble and critique a wide range of narratives, perspectives and findings about the nexus.

Our search followed the criteria specified in Table 1. We used the following search terms to screen the Web of Science (WoS), Scopus and Google Scholar databases based on Title, Abstract and Keywords: “climate change and conflict” OR “climate conflict” OR “climate violence” OR “climate security” OR “climate change and terrorism” OR “climate change and tension/unrest” OR “climate change and violent extremism” OR “climate change and armed conflict” AND “Lake Chad region” OR “West African Drylands” OR “African Sahel” OR “West Africa” OR “ Northeastern Nigeria” OR “ Borno State”.

**Table 1:** Inclusion and exclusion criteria and questions

Question	Criteria/description
*Question 1: Does the paper or document cover analysis of climate and conflict relations in the Lake Chad region?*	We included studies with a “climate conflict nexus” focus that cover one or more areas in the Lake Chad areas of Far North Cameroon, Northeast Nigeria, Southeastern Niger and Southwestern Chad. Studies covering Western Sahel or West African Drylands where Lake Chad is a part were included as well. Studies that did not meet our subject-matter and geographical criteria were excluded.
*Question 2: Does the study capture climate-related causes only or conflict impact on climate, or a combination of Lake Chad specific climatic and contextual issues, or the study questions or denies climate conflict relations?*	Studies were included if they reported climate-related causes only or conflict impact on climate, or a combination of Lake Chad specific climatic and contextual issues. Studies that question or deny climate conflict relations were included as well.
*Question 3: Is the study publicly available in English and published between 2007 and 2023?*	Studies were included if they are publicly available and published in English between 2007 and 2023.
*Question 4: Does the study use the term ‘Lake Chad’ in any way in its title, abstract, keywords or in the entire text?*	While there are various ways authors refer to the Lake Chad region, we included studies where the term ‘Lake Chad’ is mentioned, either explicitly or implicitly, at least once in the title, abstract, keywords or in the entire text, excluding the reference list. "Explicitly" here include studies that directly mention "Lake Chad" by name. "Implicitly" include those that cover the region without using the specific term "Lake Chad”.
*Question 5: Is the study accessible through electronic media (either by an open access or subscription only platform or both) to readers from various backgrounds?*	Studies were included if they are accessible through electronic media (either by an open access or subscription only platform or both) to readers from various backgrounds.
*Question 6: Does the study list or explain the methodological approach (es) used to generate evidence on ‘climate conflict’ relations?*	Methods that authors use in climate conflict studies are diverse. As such, we included studies that used longitudinal and/or cross-sectional surveys, quantitative and/or qualitative methods and/or simulations or scenario design. We also included those that are based on case studies, systematic reviews and/or context-specific indicator approaches. Studies that have not undergone peer-review, such as thesis, opinion pieces, newspaper articles and social media viewpoints were carefully screened for their correctness and included afterwards. Anecdotal evidence, personal observations and testimonials were triangulated and verified before inclusion.

‘Climate change’ often manifests in various ways in the Lake Chad region – variability, extremes, shocks and anomalies, and often operationalised as temperature, rainfall, floods or droughts. Conflict also manifests in different ways - encompassing violence, tension, war, terror, unrest, crime, protest, riot, violent extremism, kidnapping and murder (Cao *et al*.*,* 2021). As such, we used these variety of keywords in multiple combinations to capture the fullness of our subject matter and to run additional searches.

The search process covered the period from 2007 to 2023 (last access: 22 November 2023), which is the period when issues about climate security and conflict in the Lake Chad region became markedly pronounced as subjects of growing public concern, especially following several episodes of Lake Chad water fluctuations, escalation of Boko Haram attacks and presence of multiple Lake Chad related agendas in COP meetings (i.e. the Conference of Parties to the United Nations Framework Convention on Climate Change). The search process resulted in a collection of 102 articles that advance or question climate conflict links and that referred to the Lake Chad region following the criteria outlined in Table 1. We note that the inclusion of articles written only in English language presents a limitation, given that French is also a common language in countries around Lake Chad region. This limitation means future research could include multilingual sources to ensure a more comprehensive coverage.

Using the analytical strategy outlined in Table S.1 (see Supplementary Material), we Identified and evaluated diverse perspectives across all 102 selected articles, summarising the stances of different authors. Moving beyond mere aggregation or description of existing literature, we evaluated distinct narratives across studies (e.g., how the nexus is understood; how the nexus has evolved with ongoing changes in climate; how climate dynamics intersect with different phases of conflict, from latent tensions through escalation to open violence and war). Using this strategy enabled us to better understand what is known, unknown and what we need to know.

## Results

Here, we map the characteristics of Lake Chad climate conflict studies (Section 3.1) to show (i) the categories and types of studies reviewed; (ii) the scope of disciplines covered; (iii) the variety of methods used; (iv) the spatial scope of the studies; and (v) the climate indicators, conflict types, natural resource types and contextual factors captured in the publications. Then follows a description of what we know about the nexus of climate and conflict in the Lake Chad region (Section 3.2). We use our findings on ‘what we know’ about the nexus to inform our discussion about what ‘we do not know and need to know’ in Section 4. Throughout this article, the phrasing 'what we know', ‘what we do not know’ and ‘what we need to know’ is repeatedly used in the context of the collective knowledge synthesised from our 102 sources. Accordingly, “we” refers to the broader academic community or researchers studying climate-conflict linkages in the Lake Chad region.

### Characterising climate conflict studies that focused on the Lake Chad Region

**Fig. 1:**
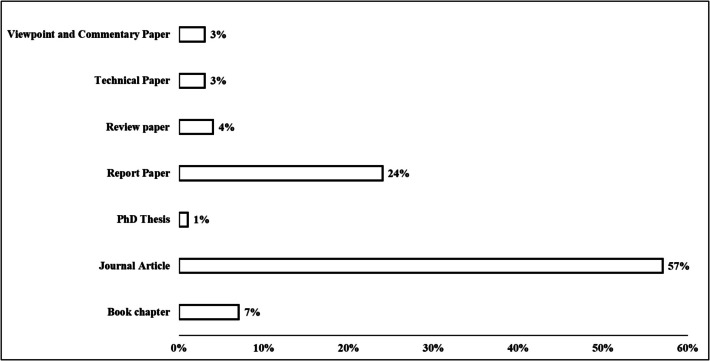
Distribution and proportion of Lake Chad climate conflict publications in our collection

Figure 1 presents the categories of studies we reviewed, covering: journal articles (57%) which present more detailed views, emphasising complexity and cautioning against oversimplification; report papers (24%) which are primarily from practitioner organisations such as the United Nations and civil society organisations generally framing climate change as a "threat multiplier," echoing its potential to exacerbate vulnerabilities and instability; book chapters (7%) which comprise in-depth climate conflict analyses within collective volumes; and others such as review papers (4%), technical papers (3%), viewpoint and commentary papers (3%) and PhD theses (1%). Technical papers in our collection offer technical reviews and are unique by their focus on technical details (e.g., lake fluctuations and rainfall variability) and provision of empirical evidence on indirect links to conflict. Relatedly, viewpoints and commentary papers reveal the authors’ personal opinions, experience and interpretations of climate conflict relations, aligning with broader geopolitical narratives and interests

The publications we reviewed are diverse in their disciplinary roots, primarily crosscutting human geography, peace science, political science, and earth and environmental sciences. The journal articles are mostly from journals such as *Regional Environmental Change*, *Sustainability*, *Environmental Science & Policy*, Political Geography, *Global Environmental Change*, and *Geopolitics*.

Figure S.1 (see Supplementary Material) shows the variety of methods – i.e., four distinct method clusters – that the publications used. These are (i) descriptive methodologies (52%): covering observational studies, surveys and case studies; (ii) mixed methods (36%): collection and analysis of both quantitative and qualitative data; (iii) exploratory methods (9%): use of brain-storming sessions, workshop discussions, interviews with experts and online surveys; and (iv) statistical and quantitative methods (2%): use of mathematical models to detect patterns, correlations and statistical significance.

All publications had a geographical focus (see Fig. S.2 in the Supplementary Material) as we intended. In delineating the publications’ spatial scope, three distinct categories emerged, each reflecting a different level of geographical specificity. First are the publications exclusively centered on the nexus of climate and conflict within the geographic boundaries of the Lake Chad basin (61%). Second are those that, although centered on the Lake Chad region, also extend their focus to cover other locations within the broader African region (sub-Saharan Africa, 2%; West Africa, 5%; West African Sahel, 4%; and the wider Africa, 13%). Publications in this second category explored the interconnectedness of climate and conflict issues, drawing comparisons and contrasts between the evidence in Lake Chad region and other African contexts. Third are those that focused on specific sub-regions or smaller parts of the Lake Chad region (15%) such as Northeast Nigeria in Borno State.

Rainfall (8%), temperature (3%) and a combination of the two (88%) represent the common climatic factors mentioned by the authors (Fig. S.3). Consequential events, such as floods and droughts, are perceived as tangible outcomes of extremes in temperature and rainfall in the area. Table 2 provides a summary of the variables (climate indicators, conflict types, natural resource types and contextual factors) that authors included in their study of climate conflict in the region.

**Table 2:** Climate indicators, conflict and natural resource types, and location-specific factors that authors use in Lake Chad climate conflict studies

Climate indicators	Conflict types	Natural resource types	Contextual factors
Temperature and rainfall (Busby *et al.*, 2011; Okpara *et al*., 2018; Wakdok and Bleischwitz, 2021; Daoust and Selby, 2023; Ehiane and Moyo, 2021)	**Herder-farmer conflict** (Brottem, 2016; Frimpong, 2020; Regan and Kim, 2020; Skah and Lyammouri, 2020; Larémont, 2021; Buba, 2021; Nagabhatla *et al*., 2021; George *et al*., 2021; Oyekanmi, 2022)	**Water and land resources combined** (LCBC, 2018; Vivekananda, 2020; Skah and Lyammouri, 2020; Larémont, 2021; Nagabhatla *et al*., 2021; UNDP, 2021; Granguillhome *et al*., 2021; LCBC, 2022; Faborode, 2022)	**Poor governance, poverty, marginalisation and corruption** (Adelphi, 2018; Vestby, 2019; Vivekananda *et al.*, 2019; Fisker, 2021)
Temperature (Namasaka, 2015; Okpara *et al*., 2017; Maharana *et al*., 2018; van Weezel, 2019; Skah and Lyammouri, 2020; Osuoka *et al*., 2022)	**Rebel conflict – Boko Haram/ISWAP** ( UNDP Ocha, 2018; LCBC, 2018; van Weezel, 2019; Griffin, 2020; Varin, 2020; Fisker, 2021; UNDP, 2021; Ani and Uwizeyimana, 2020; Sampaio, 2022)	**Water** (Okpara, 2016; Rudincová, 2017; Okpara *et al*., 2017, 2018; Frimpong, 2020; Fisker, 2021; Jedwab *et al*., 2021; Ehiane and Moyo, 2021; Oke, 2022; Ruppel and Funteh, 2023; Galli *et al*., 2022)	**Environmental dynamics and lake water fluctuation** (Cabot, 2017; Okpara *et al*., 2017; Scheffran et al., 2019; Griffin, 2020; Daoust and Selby, 2023; Oyekanmi, 2022)
Rainfall (Gao *et al*., 2011; Asah, 2015; Papaioannou, 2016; Nagabhatla *et al*., 2021; Gupta *et al*., 2021; Akinyetun and Ogunbodede, 2023)	**Communal conflict** (Lenshie *et al*., 2022; Newman *et al*., 2023)	**Land** (Brottem, 2016; Nagarajan *et al*., 2018; Sánchez, 2020; GEOGLAM (Global Agricultural Monitoring), 2020; Isah and Bitrus, 2021)	**Social, humanitarian and population pressures** (Obioha, 2014; UNFPA, 2017; Venturi and Barana, 2021; Mondo Internazionale, 2022)

We show in Fig. S.3 (see Supplementary Material) that temperature and rainfall were used separately or in combination as proxies for climate (change and variability). To link climate to conflict, authors used one or more types of conflict. The common ones are farmer-herder conflicts, inter and intra-communal violence, extremist insurgency (linked to the Boko Haram and Islamic State of West Africa Province sects), ethnic conflicts, water disputes, and political conflicts.

Authors recognise the significance of location-specific contextual factors, covering socioeconomic (e.g., population pressure, deficiency in governance, corruption, livelihood underperformance, weak social and financial assets, hike in food prices) and environmental variables (e.g., scarcity or abundance of land, forest or water resources) in their analyses. In doing this, they convey evidence depicting either a direct or an indirect link between climate and conflict in the presence of pre-existing factors. In addition, we note an evolution in narratives over time, with earlier literature (before 2012) emphasising more deterministic climate-conflict relations, while recent studies offer more nuanced, multi-factor and multi-causal explanations.

### What do we know about the nexus of climate and conflict in the Lake Chad region?

Here, we first consider why the publications we reviewed focused on the Lake Chad region. One reason is because the region has experienced significant environmental changes, including shrinking water levels and desertification, which historically have led to different conflict types. Another is that the geopolitical significance of the region, with multiple countries sharing its borders, makes it an important ‘testbed’ for understanding regional and transboundary dynamics of climate conflict relations. Knowledge from studies of climate conflict in the region is shown as useful to policymakers, researchers and humanitarian organisations working to address climate impacts and prevent conflicts (Vivekananda *et al*., 2019).

Next, in considering how the nexus is understood and presented, a sizeable body of studies portray the nexus as one that revolves around a single net climatic event (Eberle *et al*., 2020; Fisker, 2021) or as one enabled by conditional forces shaping societal vulnerability (e.g., Vivekananda *et al.*, 2019; Bressaglia and Martone, 2020; Skah and Lyammouri, 2020; Frimpong, 2020). In relation to the former, we show here three example narratives and references depicting conflict outcomes of a specific climatic event or stressor.

*……a 1 degree increase in temperature is associated with a 54% increase in conflict probability in areas that are home to herders and farmers, and a 17% increase in conflict in other areas* - Eberle *et al.* (2020)

*......climate-related stresses experienced in terms of hot temperatures provide a backdrop for the recruitment and mobilisation of extremist groups, leading to heightened security risks* – LCBC (2018)

*……a positive temperature anomaly of one standard deviation (higher-than-usual temperature) is associated with 17.6% points increase in the yearly number of conflict events taking place especially in cropland zones during growing seasons* – Fisker (2021)

Regarding narratives on societal vulnerability, we found that authors portray the nexus as one that is deeply rooted in contextual vulnerability forces which vary widely spatiotemporally within and across societies and sometimes with unpredictable spillover effects. Narratives and references showing example intervening (conditional) vulnerability forces in climate conflict relations include the following:

*…..the ongoing conflict in the Lake Chad region can be seen as a manifestation of long years of suboptimal territorial underdevelopment and climate chaos* -Tayimlong (2020)

*.........absence of effective strategies to adapt to climate change increases vulnerability, potentially triggering conflicts* – Frimpong (2020)

*......unequal distribution of the impacts of climate change have deepen economic disparities, fostering resentment and conflicts over limited economic opportunities* – LCBC (2018)

*........insufficient collaboration among countries in the Lake Chad region to address shared climate challenges contributes to conflicts over resource management* – UNDP (2021)

*……. the gendered impacts of climate change, particularly on women, are increasingly recognised as a potential source of conflict, shaping the dynamics of social and economic struggles –* *Tower (*2020*)*

Although “vulnerability” is mentioned with little explanation of what the term means, the largely “indirect link” premise upon which the climate conflict nexus is presented in most studies attests to the existence of multiple nexus transmission routes through vulnerability forces, including multidimensional fragility factors such as weak state capacity and authority, and weak state legitimacy (see Fig. S.4 in Supplementary Material). Notably, according to Vivekananda *et al*. (2019), the nexus reflects a continuum of multidimensional fragility forces that work through the “discomforting state of nature” (e.g., farmland degradation and lake drying) to shape the “nature and condition of the state/region” or the conflict behaviours of citizens.

Relatedly, “powerlessness” is central to both practitioner and academic understandings of the nexus. Using a language that communicates “powerlessness” invokes an acknowledgement of the deep-rooted vulnerabilities of lives and livelihoods across the region (Frimpong, 2020; Njoku, 2022). It follows that the contexts in which humans live, the boundaries in which states operate and the state of the natural environment in the region portray prevalent weaknesses and a condition of powerlessness, which according to LCBC (2018) and UNDP (2021) create conditions conducive for climate to fuel conflict outbursts.

Concerning how studies portray the roles of climate in conflict and the role of conflict in climate, we found that several authors (95%) view climate as a ‘*threat multiplier*’, ‘*conflict accelerator*’ or ‘*accelerator of relative deprivation and local grievances’*. A small set of publications (10%) emphasis that climatic events, such as floods and droughts, do not necessarily start conflicts. Rather, they play an ‘*amplifying role*’, for example when they trigger zero-sum struggles and contribute to entrenched illicit economies – which are conditions that local conflict actors leverage upon to drive and sustain violence (Nagarajan *et al.,* 2018; Onuoha, 2019; UN News, 2019; Jedwab et al., 2021). Climate is a threat multiplier when it undermines the capacity of the state to prepare for and carry out conflict prevention and national security responsibilities (UNDP Ocha, 2018).

The role of conflict on climate (e.g., through adaptation and mitigation) is often not specified, although some publications (15%) reveal that conflict in the region generates a big downward push on climate resilience, increasing vulnerability and maladaptation (Frimpong, 2020). Okpara et al. (2016), for example, indicate that conflict often undermines the capacity of individuals and groups to adapt to climate change, amplifying climate vulnerability and reinforcing climate conflict tipping and spillover impacts. Conflicts linked to terror groups often fuel environmental losses and damages, driving and raising carbon emissions and hot climates. Uncoordinated military response to terror groups harms the environment, further reinforcing extreme climatic conditions and thermal discomfort which ‘weakens the state of the natural environment’, predisposing the region to a continuous cycle of violence. Vivekananda *et al*. (2019) call this a “climate-fragility-conflict trap”. A final point to note here is that, across the Lake Chad region, climate and conflict are tied together in a vicious circle where conflict undermines the capacity to cope with climatic events, with climate making it harder to address conflicts and promote peace (Nagarajan et al., 2018; Singh, 2022). While our inclusion criteria deliberately sought studies that question or deny climate-conflict relations in the Lake Chad region (see Table 1), our analysis revealed a nuanced picture pointing to a lack of direct refutation. We found no studies that explicitly refuted climate-conflict linkages in the region. This absence itself suggests a valuable finding, reflecting the current state of knowledge in this area.

Switching to how the nexus has evolved with ongoing increase in regional climatic stress where temperatures are rising 1.5 times faster than the global average, more recent studies reveal: heightened rate of forced migration (UNHCR, 2016; Akubor, 2017; IOM, 2019; Kamta *et al*., 2021; Lamarche, 2022; Tower, 2022); more land use changes (Nwilo *et al*., 2019; Nwilo *et al*., 2020); and hike in food prices (Blankespoor, 2020; Pham-Duc *et al.,* 2020; Olowoyeye & Kanwar, 2022). Multiple conflict types (e.g., gender-based violence and violence against children) are happening simultaneously with communal battles and land violence increasing by more than 50% since the emergence of Boko Haram conflict in 2009 (Cole et al., 2016; Brechenmacher, 2019; UNDP, 2022). Conflict fatalities peaked in 2014 and 2015 to around 1,000/year (ACLED, 2022) and regional conflict spillover under hot temperatures and resource scarcity has continued to reinforce interactions between different conflict types, further strengthening the nexus (Okpara et al. 2016). This spillover is enabled by transnational armed groups and militant networks operating across Nigeria, Chad, Cameroon and Niger, exploiting porous borders to fuel overlapping conflict types and sustain violence and illicit economies.

As has been echoed in the environmental security literature, empirical evidence and anecdotal narratives on the nexus often differ and sometimes misrepresent the nexus, leading to flawed policies (Mach *et al*. 2020). However, this is not the case in the Lake Chad region where both forms of knowledge (when integrated) can provide a rich insight about the nexus (Skah and Lyammouri, 2020). While the authors advancing scientific evidence rely on rigorous research methodologies, including data analysis and peer-reviewed studies to draw conclusions, studies based on anecdotal reports and stories capture the lived experiences and narratives of individuals and local communities, offering qualitative perspectives on the nexus. The former offers a more systematic and somewhat reliable quantitative understanding of the nexus patterns, whereas the latter enriches the narrative with contextual nuances that capture the human and locational aspects of the nexus. In cases where certain structural determinants of the nexus – such as fractured social and political systems, poverty and resource depletion - are difficult to quantify in empirical models, anecdotal narratives come in to help provide more insightful ways of pinning down the breadth and scope of the determinants and the overall nexus dynamics.

Perceptions and responses regarding how to deal with climate conflict concerns differ across the various publications we reviewed. Some (e.g., Okpara *et al*., 2018; Vivekananda *et al.*, 2019; Griffin, 2020; Frimpong, 2020; Fisker, 2021; Jedwab *et al*., 2021) emphasised the integration of climate action, peace action and water action, whereas others (e.g., Adelphi, 2018; Vestby, 2019; Skah and Lyammouri, 2020) suggested interventions that target behavioural change, building of stronger environmental governance and human rights institutions, and maintaining sustainable access to jobs and income. Ultimately, breaking the nexus requires differentiating between specific conflict- versus climate-induced vulnerabilities and related socioeconomic crises fuelling the nexus (Griffin, 2020). Doing this is particularly challenging because the climate conflict nexus often overlays other nexus dynamics at play – namely the humanitarian-development-peace nexus, thus making it even harder for authors to identify effective strategies to address climate conflict stressors and local peace needs all at once.

For these reasons, especially considering the urgency to tackle the climate conflict crisis, UNDP (2021, 2022) posits that promoting integrated and multidisciplinary approaches is crucial. In fact, according to Mercy Corps (2017), multidimensional approaches that combine both science-based and traditional response/resilience mechanisms are necessary to foster effective locally-relevant and community-led resilience – not only to operationalise locally-led conflict transformation ideals, but also to identify effective responses specifically aimed at preventing and compensating for climate and conflict induced losses and damages. Notably, solutions that address the nexus in a holistic manner are emerging, but clear impacts are hard to pin down in the Lake Chad region.

From the interdisciplinary review and results presented in this section, Table 3 provides a summary of what we know about the nexus of climate and conflict in the Lake Chad. This is further conceptualised in Fig S.4 (see Supplementary Material): climate and conflict are tied together as a self-reinforcing cycle, with livelihood vulnerabilities and fragility in governance sustaining the cycle and feedback loop, undermining the region’s capacity to adapt and respond effectively.

**Table 3:** Summary – what we know

Themes	Summary: What we know
How the nexus is understood and presented	The nexus is understood to revolve around, and is shaped by, a single *net climatic event* or as one enabled by *conditional forces* linked to societal vulnerability and the powerlessness or weakness of the state and citizens. The nexus dynamics is rooted in the “weak state of the natural environment” and “a highly vulnerable status of the state”, producing climate conflict outcomes that vary widely across the region. Evidence challenges the ‘*climate centric*’ and ‘*denial claim*’ discourses espoused in Okpara et al. (2016), suggesting that conflict in any guise is **not a direct response** to climatic events. Climate-sensitive asset holdings derived from unstable land and water-based activities often serve as a medium through which climatic events undermine livelihoods, offering a pathway to conflict.
The role of climate in conflict and role of conflict in climate	A sizeable body of studies portray climate in the Lake Chad region as a ‘*threat multiplier*’, a ‘*conflict accelerator*’ and an ‘*accelerator of deprivation and local grievances*’. Climatic events do not readily start conflicts. However, they can propel early conflict onset, stretch conflict duration over multiple timescales and across societies or amplify conflict severity. Conversely, conflict weakens the resilience of communities and institutions to respond to climatic events; it weakens the capacity of governments to initiate and execute climate-compatible development. Across the Lake Chad region, climate and conflict are tied together in a vicious circle where conflict undermines the capacity to cope with climatic events, with climate making it harder to address conflicts and promote peace.
How the nexus has evolved with ongoing increase in regional climatic stress	With temperatures rising 1.5 times faster than the global average, the region has experienced heightened rate of conflict-induced forced migration, more land use changes and hike in food prices. Multiple conflict types are happening simultaneously with battles and violence increasing by more than 50% since the emergence and rise of new conflict types such as Boko Haram conflict and gender-based violence. Conflict fatalities peaked in 2014 and 2015 to around 1,000/year and regional conflict spillover under hot temperatures and resource scarcity has continued to reinforce multiple conflict types, further strengthening the nexus and worsening living conditions. Transboundary conflict spillover is a distinct characteristic of climate-conflict linkages in the Lake Chad region, enabled by transnational armed groups and militant networks operating across Nigeria, Chad, Cameroon and Niger, exploiting porous borders and citizens’ vulnerability to fuel and sustain violence and illicit economies.
Empirical evidence vs anecdotal evidence	When integrated and properly triangulated, both types of evidence can provide insightful understandings of the nexus in the Lake Chad region.
How authors link climate change across the conflict continuum or conflict cycle	A small set of studies link climate to two noticeable phases in the conflict cycle: conflict onset/emergence and violence escalation. We note that authors need to consider climate influences on other conflict phases, e.g., conflict duration, patterns of conflict spillover, de-escalation and resolution, and reoccurrence.
Perceptions and responses for dealing with climate conflict challenges	Solutions that address the nexus in a holistic manner are emerging, however clear impacts in terms of sustainable peace and climate resilience are yet to be seen.

## Discussion: What we don’t know and need to know

In reviewing studies on Lake Chad climate conflict, we observed six important knowledge clusters that convey evidence of the nexus, i.e., “what we know” (see Table 3). While our results are somewhat consistent with other systematic reviews of published work in the field of climate conflict and security (e.g., see Scartozzi, 2021), we do not claim that Table 3 provides an exhaustive summary about the nexus for the Lake Chad region. The increase in climate conflict publications on this region indicates a growing interest in studying the material consequences of climatic events on livelihoods, the economy and on land and water resources, yet it remains the case that conflict is often emphasised but rarely assessed as a continuum.

Conflict in the Lake Chad region manifests as a *continuum* that stretches from latent conflict to visible conflict and armed violence (Griffin, 2020; Frimpong, 2020; Singh, 2022). It is experienced in different forms and types, ranging from banditry, kidnapping and violent extremism to gender-based violence, farmer-herder conflict, land and water communal conflict and rebel violence (van Weezel, 2019; Buba, 2021; Nagabhatla *et al*., 2021; Oyekanmi, 2022; Lenshie *et al*., 2022; Newman *et al*., 2023). These conflict types are dynamic and sometimes interacting and reinforcing one another spatiotemporally (Skah and Lyammouri, 2020). They interact in ways that undermine the capacity of regional authorities to prioritise, pursue and achieve conflict transformation.

In considering how authors link climate across the conflict continuum and whether climate affects the conflict cycle in relation to conflict emergence (onset), escalation, de-escalation and conflict reoccurrence, we note that a small set of studies (10%) link climate to conflict onset (Okpara, 2016; Fisker, 2021) and violence escalation (UNDP, 2022). However, how climate influences other conflict attributes, e.g., conflict duration, patterns of conflict spillover, conflict de-escalation and reoccurrence, remains largely understudied. It follows that authors failed to account for how climate interacts with different conflict phases and cycles, and whether climatic events also introduce new forms of conflict along the conflict continuum. There is also no account of how climate impacts experienced in one part of the region produce conflict outcomes in another part.

Furthermore, the exact conflict types linked to climatic events in the region remain the subject of much debate. In the past, the focus used to be on transboundary water conflicts, but now evidence of indirect climate connection to farmer-herder conflict, rebel violence, land theft/grabbing, and cattle rustling has emerged (Lamarche, 2023). There is the likelihood that climate might affect all conflict cycles/phases in different ways, yet we do not know the effects of climate on conflict tipping and conflict economies, including how different conflict types reinforce each other under varying climatic conditions.

Many studies consider temperature and rainfall, including droughts, floods and heatwaves, as important climate indicators. However, climate is often not well conceptualised and there is a lack of interest to portray this as either a slow-onset event or a sudden-onset change. Slow-onset events, such as droughts, are easily noticeable in the Lake Chad region, alongside sudden-onset disasters, such as floods, which are more readily discernible and very destructive. These events exert different influence on conflict. For example, the psychosocial discomfort and other subjective life situations droughts and hot climates create often contribute to hostile thoughts, mental disorder, maladaptive behaviour, aggressivity and violence (Dodgen *et al*., 2016; Hayes *et al*., 2018; White *et al*., 2023; Savelli *et al*., 2023). As a consequence, studies focusing on the region should consider delineating climatic conditions along slow-onset effects and sudden-onset disasters in ways that account for country specific constraints and impact pathways.

There is currently little knowledge about how the nexus differs across Lake Chad countries and communities. It seems reasonable to assume that Chad and Nigeria, where a relatively large portion of the Lake Chad waters is currently located, might reflect a somewhat positive signal of climate conflict relations in the presence of economic and political exclusion for example, compared to Cameroon and Niger. It also seems that some areas facing extreme droughts and desertification (e.g., Far North Cameroon) might experience less conflict if the nexus amplifies the need for survival, cooperation, delivery of humanitarian aid and other development assistance. Similarly, pastoralists might be highly vulnerable than other livelihood groups in terms of climate-induced aggressive behaviours and willingness to fight – their migratory lifestyle could mean they have poor social networks and income strategies which could fuel their motivation to fight. Diverse patterns of and exposure to climate and conflict across the region might indicate a need for differential intervention measures based on disparate local contexts. These propositions are based on Okpara (2016) whose work on climate, conflict and livelihood vulnerability acknowledges the wider regional socioeconomic disparities in which the nexus could exert differential impacts and responses.

Notably, consideration of differential nexus patterns and impacts suggests a need to also account for heterogenous fragility. Our assessment shows that authors missed to ask the question of how fragility (assessed in terms of lack of state legitimacy, capacity and authority) shapes ‘group identity/solidarity’ and differential nexus patterns and impacts. The Lake Chad region is characterised by a fragmentation of countries inhabited by groups with unique national identities who view the Lake Chad's resources as essential assets worth capturing, controlling and plundering (Gao *et al*., 2011; Maza *et al*., 2020). Groups (e.g., farmers, fishers and herdsmen) perceive their struggles for economic gains as a zero-sum game (Okpara *et al*., 2017). Climate conflict impact is experienced differently by these groups, and their level of tolerance and vulnerability vary depending on their asset holding. The mentality of "*it is our turn or right to control and rule*" is prevalent in the region (Ewi and Salifu, 2017). Groups do not perceive the authorities governing the assets in the region as legitimate. This often motivates unhealthy group formation and rebellion, undermining state authority and the rule of law (International Crisis Group, 2024). The Boko Haram sect, for example, does not accord legitimacy to the Lake Chad regional governments. This lack of legitimacy and the view of the region as a resource to be plundered contribute to the crisis of climate conflict in the area (Maza *et al*., 2020). Further, the inability of the states in the region to perform basic functions, such as supporting community adaptation to climate change, providing climate services, ensuring the security of citizens, and developing infrastructure, hinders the effectiveness of climate and peace actions. This lack of state capacity is compounded by a lack of state authority and legitimacy in the face of climatic stress, contributing to sporadic clashes, violence and rebel attacks. Insecurity is further compounded by insufficient household income, an underdeveloped private sector and an economy that is heavily dependent on climate-sensitive assets and activities. This feedback loop contributes to diminishing government revenues, further shrinking state capacity and authority and exposing countries in the region to socioeconomic and climatic shocks.

Some methodological approaches, such as big data analytics and machine learning, have yet to be employed in studies investigating climate conflict relations in the region. Others, such as Artificial Intelligence, geospatial techniques (e.g., geographic information systems and remote sensing), and predictive analytics are yet to be utilised, suggesting that our knowledge of how these methods could enhance understanding of climate conflict in the area remains unclear. Additionally, "complex systems" techniques such as futures thinking, scenario design, and multifactor mapping, which can help unpack important dimensions such as the role of cognition (in relation to human thermal discomfort, mental health, and other cognitive dimensions) and maladaptation in climate conflict, have not been widely adopted. Geospatial techniques, for example, provide exceptional capabilities in spatial analysis and visualisation, offering a valuable perspective for examining the different geospatial impact dimensions of climate conflict dynamics (Kamel Boulos and Wilson, 2023). Comparative case studies, historical analyses, modelling and scenario designs enable researchers to uncover nuanced contextual factors, historical trajectories and potential future scenarios, thereby enriching the depth and breadth of climate conflict analyses (Ide, 2017).

The apparent omission of these methodologies in the publications we reviewed highlights a crucial gap in methods as well as a promising opportunity for future methodological innovation in climate conflict research in the Lake Chad region. Integrating advanced techniques can offer a more comprehensive and nuanced understanding of the intricate connections between climate and conflict, and ultimately contribute to the refinement of strategies for conflict prevention, mitigation and sustainable development in the area. Taken together, Table 4 provides a summary of what we don’t know and need to know about the nexus of climate and conflict in the Lake Chad region.

**Table 4.** Summary: what we don’t know and need to know

Since conflict manifests as a continuum, we don’t know (i) how climatic events affect all stages of the conflict cycle and continuum; (ii) whether climatic events also introduce new forms of conflict along the conflict continuum; (iii) how climate impacts occurring in one part of the region produce conflict outcomes in another part; (iv) whether climatic events influence how different conflict types interact and reinforce one another; and (v) the role of climate in conflict tipping, conflict spill over and the rising conflict economies across the region.	
Climate is often not well conceptualised and there is no evidence how it is delineated either as a slow-onset event or a sudden-onset crisis in a manner that reflects country/regional specificity.	
There is little knowledge about how the nexus differs across the Lake Chad countries and communities, and moreover less is known about the differential impacts of double exposures to climate and conflict on different livelihood groups.	
Any consideration of differential nexus patterns and impacts would need to take account of heterogenous fragility, yet authors missed to ask the question of how fragility (assessed in terms of lack of state legitimacy, capacity and authority) shapes ‘group identity/solidarity’ and differential nexus patterns and impacts.	
Certain beneficial methods (e.g., big data analytics, machine learning, geospatial techniques and predictive analytics) have yet to be employed in studies investigating climate conflict relations in the region, suggesting that our knowledge of how these methods could enhance understanding of the nexus in the area is limited.	

Beside unpacking what is known and unknown about Lake Chad climate-conflict issues, our review shows notable interdisciplinary variations in evidence, mainly due to diverse epistemological approaches. For example, climate science prioritises quantitative modelling of long-term trends, while social science emphasises qualitative, community-level evidence. Fragmented evidence spans narratives concerning single net climatic events, contextual vulnerability, fragility and powerlessness, the role of conflict in climate and climate in conflict, evolution of the nexus over time under hot climates, and storylines on responses and solutions. This disciplinary fragmentation of evidence presents challenges for creating a unified understanding, but also opportunities for rich, multifaceted knowledge integration and co-creation. To bridge these disciplinary divides and integrate fragmented evidence, prioritising transdisciplinary approaches, stakeholder engagement, and integration of diverse data types and methods will be essential (see Pacillo *et al*., 2024). This approach can foster collaborative learning and cross-sectoral exchanges, ultimately creating a more comprehensive understanding of climate-conflict dynamics in the region. Knowledge co-creation can help integrate fragmented evidence about the nexus, fostering a unified, coherent, and verifiable body of knowledge. This integrated, co-created knowledge base can support joint climate and peace initiatives and wider transformative change across the region. However, we note that this process may face challenges, such as reconciling different methodologies and epistemologies across disciplines. Despite the challenges, the benefits of a more holistic understanding of the climate-conflict nexus in the Lake Chad region make the pursuit of interdisciplinary and knowledge co-creation an essential endeavour.

## Conclusions and ways forward

We reviewed published work on the nexus of climate and conflict in the Lake Chad region to identify what is known and what remains unknown about the nexus. Specifically, our analytical strategy traced diverse narratives and patterns, developing an integrative framework that captures how the ‘Lake Chad’ climate-conflict nexus is understood (see Fig. S.4), making this article a key resource for advancing climate-conflict knowledge both within the region and more broadly.

We show six key areas of knowledge that provide evidence of the nexus (i.e., “what we know”), which are summarised in Table 3. Additionally, we outlined in Table 4 what we do not know and what we need to know about the nexus. Table 3 details regional conflict spillover which has continued to reinforce multiple conflict types under hot temperatures in Lake Chad (resurgence of resource-based conflicts alongside ongoing insurgencies). This is a phenomenon particular to the Lake Chad region with temperatures rising 1.5 times faster than the global average. Similarly, Table 4 illustrates specific ‘unknowns’ peculiar to Lake Chad, such as how climate impacts in one part of the region produce conflict outcomes in another, revealing trends not previously identified in general climate-conflict reviews or in other climate-conflict hotspots. Both Tables present results that are distinctly characteristic of the Lake Chad region.

The specificities of existing evidence on the Lake Chad region are shown in Fig S.4 (Supplementary Material) – demonstrating that climate and conflict are tied together as a self-reinforcing cycle where vulnerability and fragility forces are seen as sustaining the region’s climate-conflict feedback loop. This trend is significant considering that Lake Chad spans four countries with varied vulnerabilities, varied conflict tipping risks and spillover dynamics, making it sometimes harder to identify effective strategies that can tackle regional climate and conflict concerns all at once. This represents a dynamic not commonly observed in other climate-conflict hotspots.

Taken together, our review provides a nuanced, region-specific analysis of the climate-conflict nexus in Lake Chad, moving beyond general discussions to offer actionable insights for regional stability and future research directions. The Lake Chad case supports the general evidence that climate change often exacerbates existing conflicts and create new tensions in fragile places. Relatedly, our narratives and findings diverge from broader debates on what is unknown about climate-conflict. For example, the transboundary nature of Lake Chad suggests we need to know how climate impacts occurring in one part of the region produce new or reinforce old conflicts in another part; and with conflicts manifesting as a continuum, we need to know how climatic events affect different conflict phases and cycles simultaneously. These are significant elements of our findings that are often not captured in other climate-conflict hotspots.

Ultimately, the value of this article lies in our argument about the need to create a structured, interdisciplinary knowledge base on climate conflict to foster collaborative learning and cross-sectoral exchanges in the Lake Chad region. To support this endeavour and advance ways forward, we ask: *How do we best utilise the available evidence, and how do we make the unknown known to support efforts towards joint climate resilience and sustainable peace?*

Whilst it is difficult to address these questions without involving critical stakeholders, we contend that to maximise current evidence could imply using what we know to drive solutions that are sensitive to the interplay of climate and conflict (e.g., peace-centred and climate-compatible development through livelihood rehabilitation and environmental stewardship). Although no metrics exist for identifying solutions that are most promising, understanding how policymakers are utilising existing evidence and the best ways to collaborate with them to creatively address climate and conflict threats simultaneously, is essential. Policymakers are sometimes busy fixing administrative systems, but they also need to demonstrate strong political will, making the necessary choices to drive the required solutions. In addition, utilising current evidence could also mean recognising the positive aspects of climate and conflict, such as how climate could serve as a catalyst for prosperity and peacebuilding efforts, including how conflict could create opportunities for peace education, collaboration and policy development.

To *make the unknown known*, there is a need to combine methods, theories and transdisciplinary approaches from multiple knowledge systems and *'ways of knowing'*. This can happen through collaborative work between environmental and social scientists, mediators, peace actors, humanitarian agencies and policymakers. Encouraging 'knowledge co-creation' (e.g., by using citizen labs) can help integrate fragmented knowledge into a unified, coherent and verifiable body of evidence.

In sum, transforming any climate and conflict afflicted fragile region such as the Lake Chad region will require a combination of social-ecological actions that are short and long-term in design and that span multiple generations. Certain pivotal events, such as changes in leadership and financing for climate and peace, could present opportunities for local actors and governments to implement needs-based strategies and achieve "quick wins", leading to visible, immediate, rapid and significant regional transformations.

## Supplementary Information

ESM 1 (DOCX 137 kb)

## Data Availability

Not applicable
