# Supplementary material for: The nexus of climate and conflict in the Lake Chad Region: what we know, don’t know and need to know
Source: Clim Change. 2025 Oct 14;178(10):185. doi: 10.1007/s10584-025-04024-0 (PMC12521271; doi:10.1007/s10584-025-04024-0)
Supplement: Supplementary file 1 — (DOCX 137 kb) [file 10584_2025_4024_MOESM1_ESM.docx]

Electronic Supplementary Material

Online Appendix for the article titled: “**The Nexus of Climate and Conflict in the Lake Chad Region: What We Know, Don’t Know and Need to Know**”

Table S.1. Analytical approach: data extraction, retrieval of evidence and evaluation

| *Surface descriptor* | This relates to the title, abstract and keywords in the retrieved studies. |
| --- | --- |
| *Central entities and/or issues recognised* | We used the following questions: Does the study reveal why studying climate conflict matter for the Lake Chad region? How is the nexus understood? What role(s) does climate play in conflict outcomes? How does conflict affect climate? What specific climate indicators and conflict types are used? How has the nexus evolved with ongoing changes in climate? What evidence is given to show how climate shapes the conflict cycles across various stages from latent conflict to visible conflict, violence and/or war? In what ways are conflict and climate relations different across the region? |
| *Association or causality and mechanistic relationships* | We used another set of questions: What narratives depicting climate and conflict links, mechanistic relationships, pathways and impacts are presented? |
| *Normative judgements* | Where do authors stand (i.e., authors’ established position on the ‘causality’ arguments). What are the various perceptions regarding ways to respond and deal with climate and conflict threats? Any policy prescriptions? |
| *Illustrative conclusions suggesting future directions or solutions* | What are the central issues recognised as next steps? Is the study explicit about where we are headed? What opportunities are identified as ways to maximise current evidence to achieve peace and stability in the region? |


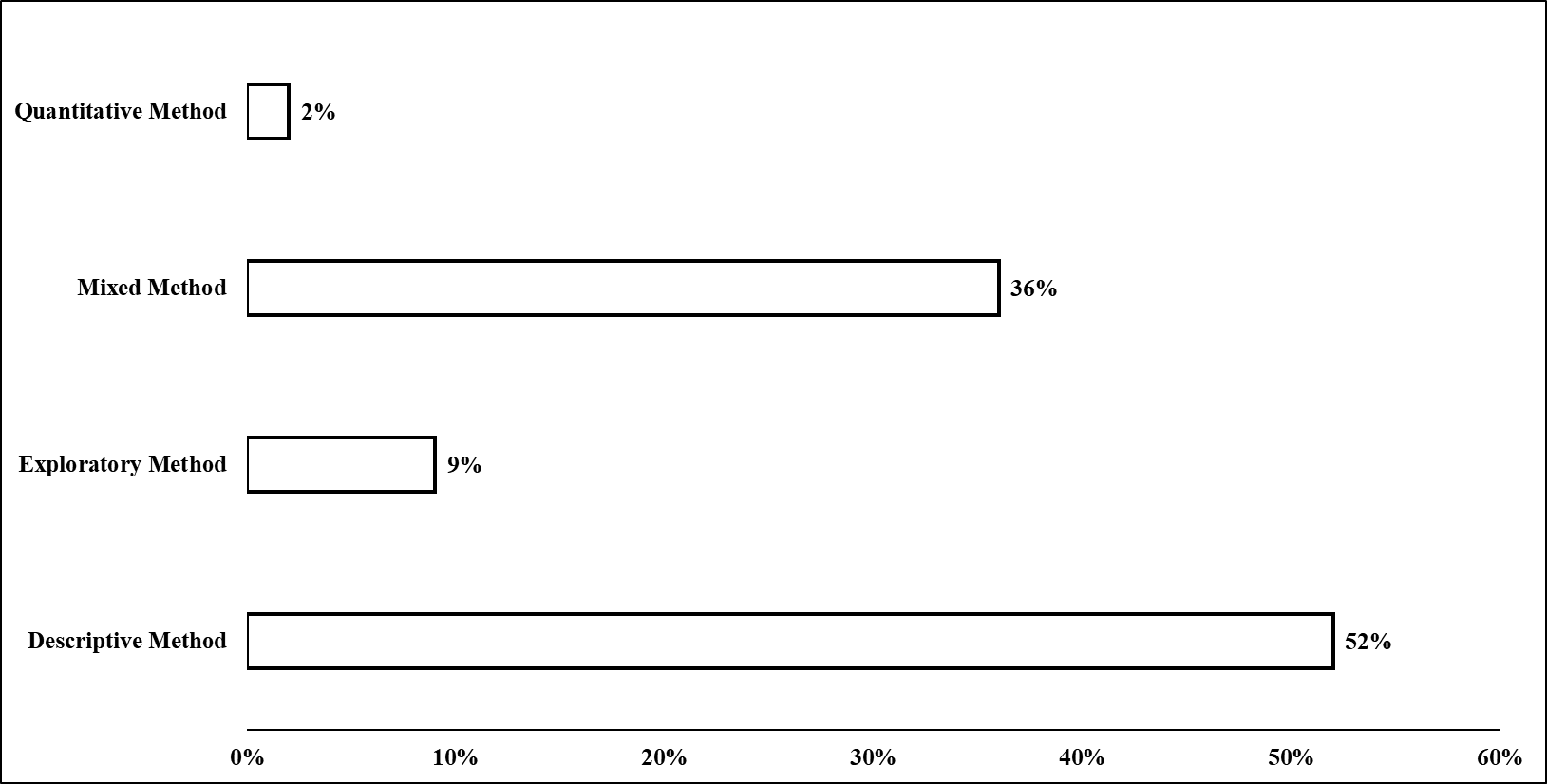


Fig. S.1: Four method clusters/categories used in the publications in our collection.


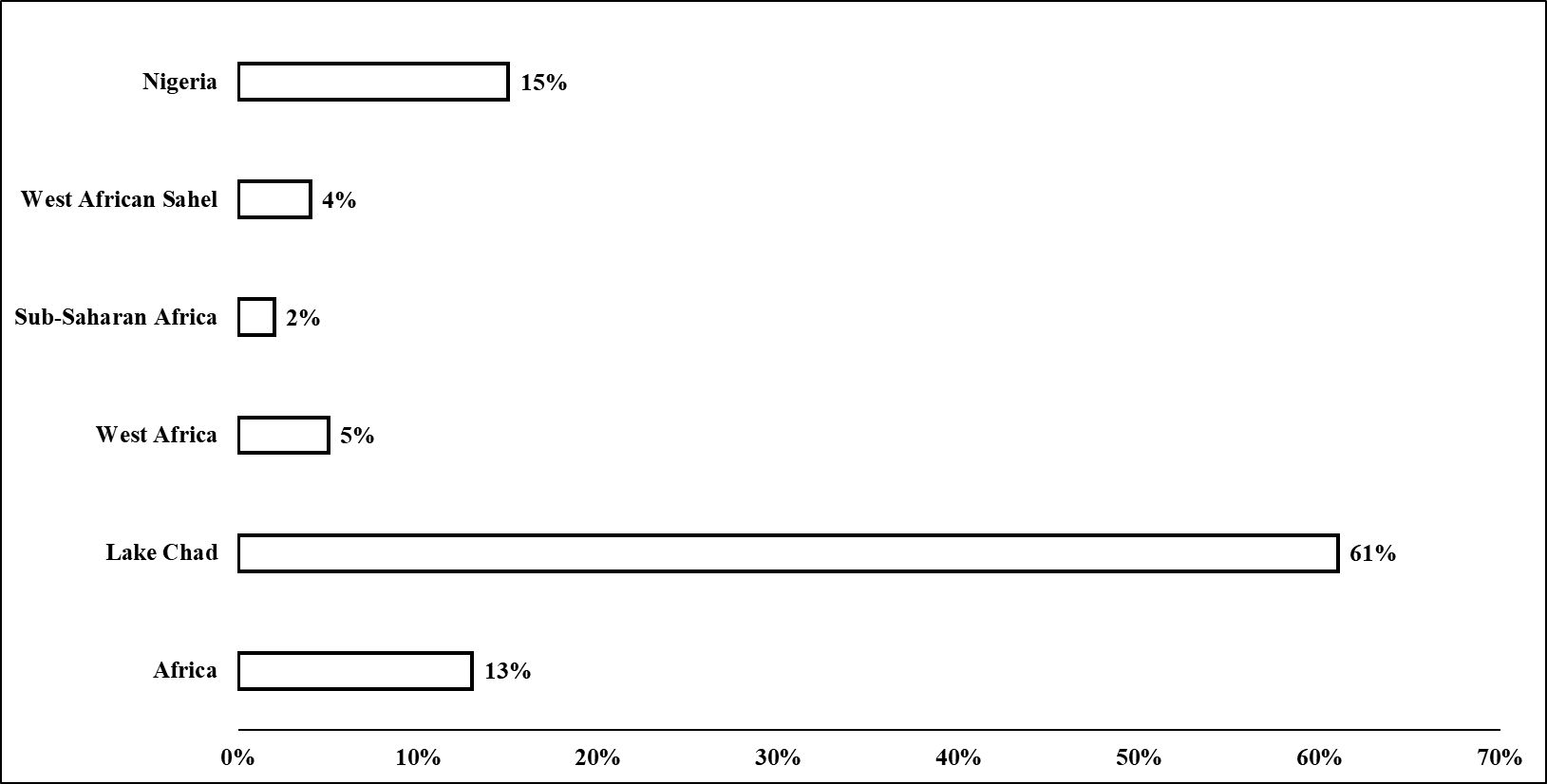


Fig. S.2: Spatial scope of the publications reviewed.


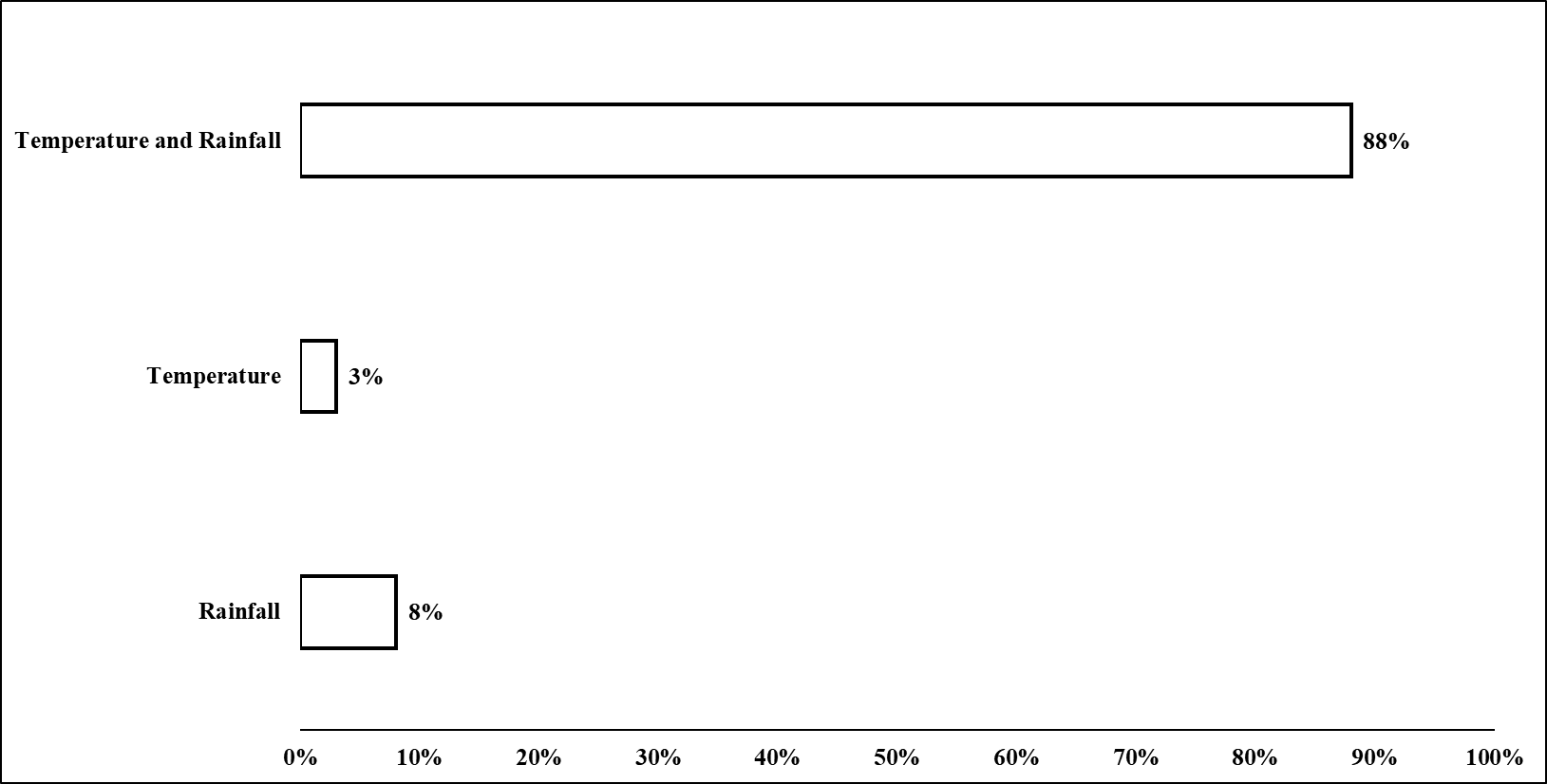


Fig. S.3: Temperature and rainfall measures are common climatic factors.


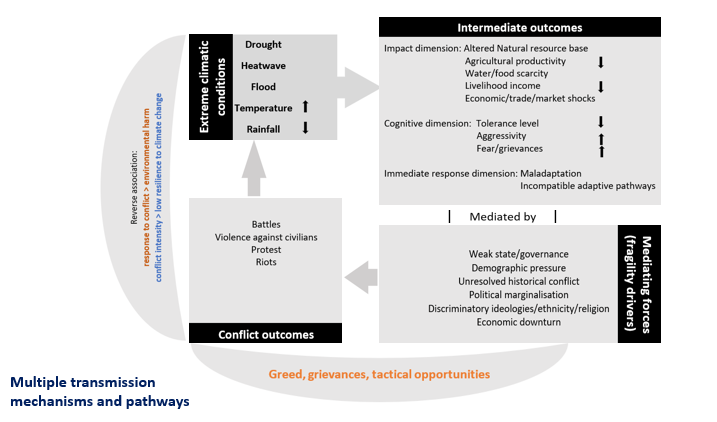


Fig. S.4. Summary of how the nexus is understood and presented in a sizeable body of studies that we reviewed.
